# School Nursing

**Published:** 1919-03-08

**Authors:** 


### THE LIFE OF A NURSE: ITS MORAL AND MATERIAL ADVANTAGES.

### III. School Nursing.

THE value to the State of the trained nurse in the working and carrying out of the various progressive schemes associated with the preservation of health and the prevention of disease is now more fully recognised, and in consequence a large field of attractive, interesting work has opened out for the trained woman of good education and wide sympathies.

One of the most important and interesting branches of national health service is that of school nursing, dealing mainly with children between the ages of five and fourteen years.

With the passing of an Act in 1907 called "The Medical Inspection of Children in Public Elementary Schools Act," which gave administrative powers to educational authorities to provide for a complete system of medical inspection in the elementary schools, there arose a new era in the life of the school child which has proved of incalculable benefit. This Act came into force in January 1908. Medical men of repute who had the interests of the public welfare at heart were appointed to organise the scheme throughout the country, and, as the work developed, city and county educational authorities appointed school medical officers for their local needs, and the services of a school nurse became a necessity.

The wide scope and importance of school nursing work will be readily understood when it is brought to mind that the broad aim of this system of medical inspection is to improve the physical, mental, and moral condition of this and of coming generations. To detect conditions which lead to incapacity, to advise and, if practicable, carry out the necessary treatment to remove or remedy the defect so as to make the pupil as efficient as possible both in school and adult life.

As an instance of the benefit to the educational career alone, the most pressing need of backward children is medical attention. The causes of mental dulness are commonly due to defective eyesight and hearing, the presence of adenoids and throat affections, or a low condition of the nervous system.

### PROFESSIONAL QUALIFICATIONS.

The professional qualification for a school nurse is three years' training in a general hospital. It is well to have a period of training in the out-patient department, in the children's ward, and the special departments such as skin diseases, eye, ear, and throat, also fever nursing, which are likely to be most useful to the chosen cause on leaving hospital. Though little actual sick nursing comes into the school nurse's life, the detection and treatment of minor ailments are some of the essential duties. The certificate of the Royal Sanitary Institute for sanitary inspectors or for health visitors and school nurses is a valuable addition to that of sick nursing, as a great deal of the school nurse's work lies in the homes of the children, and the training gives one an insight into unfamiliar problems of hygiene, dealing with the home life and social conditions of those under supervision. The syllabus for the school nurse's certificate is a very comprehensive one; besides the subjects of physiology and hygiene generally, special attention is given to hygiene of schools, air, ventilation, water supply, food and clothing, communicable diseases, home nursing, and care of infants, first-aid treatment, elementary knowledge of health statistics. One point to bear in mind is that though the matters coming under the head of hygiene appear exceedingly simple to the trained nurse, as an educator and advisor simple and lucid language is required when explaining to uneducated people and ignorant children what one may know perfectly in technical language oneself. In most of the large provincial towns the principal technical and women's colleges prepare students for the examinations by lectures

☞ Previous articles appeared on February 8 and 15, p. 435.

**School Nursing—(*continued*).**

and demonstrations from qualified teachers, or, if a student lives in a country place, a correspondence course may be arranged, the candidate going up, when ready, to the nearest centre where the examinations are held.

PERSONAL QUALIFICATIONS.

The personal qualifications usually demanded are an attractive, courteous manner, tact, sympathy, and a saving sense of humour. Patience and cheerfulness in the face of failure are worth cultivating; also a real love of people, especially "little people," and a gift of imparting knowledge are essentials. Indeed, one would well-nigh have to be an "Admirable Crichton" for the varied attainments considered necessary. Good health is essential, as the nurse is required to go out in all weathers, and there is a good deal of fatigue both in standing in schools during routine inspections and in visiting in the district after school hours. Warm, light clothing and comfortable footwear are most necessary, also good nourishing meals at regular intervals. The school nurse may either be employed in medical inspection generally or work in a special school for mentally and physically defective children, in connection with the cleansing departments, at a treatment centre for dental and eye work, or at a special school for ringworm and skin diseases. Such varied duties depend, of course, on the development of the work where one is stationed. The general routine work required is, briefly, to assist the doctors in routine inspection, to inspect the heads and clothing of school children for the presence of vermin, to detect skin diseases, ear discharges and eye affections, to ascertain whether defects recommended for treatment by the school medical officers have received due attention, and to visit children's homes in order, if necessary, to remedy faulty living conditions, keeping detailed reports of work done. Conducting health talks to parents and the older children may also be required.

PROSPECTS, OPPORTUNITIES, AND PRIVILEGES.

In towns where there is an adequate medical staff a nurse usually works with one medical officer having a certain group of schools to which visits are periodically paid. Thus she gets to know her district well, and the teachers and children have confidence in her work and welcome her visits.

In a small town or country district the school nurse may also be called upon to act as health visitor or tuberculosis visitor in connection with a Government dispensary.

The future prospects of the school nurse compare favourably with any branch of nursing. The work is not really so monotonous as it seems, and as the work develops the nurse's work will be better systematised. The work lies among healthy as well as defective children. If one takes an interest in future schemes and keeps one's knowledge up to date, supervisory posts may be available, lecturing in college on social subjects, managing children's convalescent homes, or any special branch such as mentally defective schools or homes. The moral and material advantages of a school nurse's life are the great experience gained in the knowledge of human nature and the many different character-studies one is confronted with. Facts have to be faced, and a great deal of patience and self-control is required in preventive educational work. One still finds extraordinary prejudices and superstitions prevailing, such as piercing the ears and wearing earrings as a corrective to defective vision, and the idea that the presence of head lice is an outward and visible sign of robust health. Genuine convictions are difficult to displace with scientific and rational methods, so one is forced to "make haste slowly." The necessity of living a regular, orderly life, if one is to keep fit and set a certain standard of hygienic efficiency by example as well as precept, is quite good for one's health. It is a benefit that one has regular hours, usually from nine to five, with an hour off for dinner in the middle of the day. The evenings are free, and one can with advantage take up some hobby or take up some line of recreative study such as literature, art, or music. The continuation classes for adults in the Council schools have a wide range of subjects where one can derive instruction and pleasure for a very moderate fee. Saturdays are free as a rule, and a week-end occasionally in the country or at the seaside proves a refreshing tonic after the wear and tear of crowded, dirty streets and refractory individuals. A fixed salary is given, usually from £80 to £110 per annum, with the addition of uniform, laundry, and travelling (*i.e.* car) expenses, according to the ideas of the various educational authorities. It is hoped the scale of salaries is to be revised shortly.

Holidays are given, when the schools close, three times in the year, which allow one to recuperate and enjoy the change of healthy open-air life as a contrast to combating sordid conditions. The necessity of keeping oneself fresh when doing practical social work is no less than a duty, and, though school work may appear exacting and depressing at times, one has to remind oneself it is all so tremendously worth while. We are reminded by the great writer Carlyle that "There is no kind of achievement equal to perfect health."

